# Analysis of Locking Self-Taping Bone Screws for Angularly Stable Plates

**DOI:** 10.1007/s40846-017-0279-4

**Published:** 2017-06-21

**Authors:** Radek Čada, Karel Frydrýšek, František Sejda, Jiří Demel, Leopold Pleva

**Affiliations:** 1 Department of Mechanical Technology, Faculty of Mechanical Engineering, VSB – Technical University of Ostrava, 17. Listopadu 2172/15, 708 33 Ostrava, Czech Republic; 2 Department of Applied Mechanics, Faculty of Mechanical Engineering, VSB – Technical University of Ostrava, 17. Listopadu 2172/15, 708 33 Ostrava, Czech Republic; 3 Trauma Centre, Faculty Hospital of Ostrava, 17. Listopadu 1790/5, 708 00 Ostrava, Czech Republic; 4 Faculty of Medicine, University of Ostrava, Dvořákova 138/7, 701 03 Ostrava, Czech Republic

**Keywords:** Traumatology, Orthopaedics, Locking bone screw, Angularly stable plate, Osteosynthesis, Biomechanics

## Abstract

Paper focuses on biomechanics, specifically on locking cortical bone screws in angularly stable plates used for the treatment of bone fractures in the medical fields of traumatology and orthopaedics. During extraction of titanium-alloy implants, problems are encountered in an effort to loosen some locking bone screws from the locking holes of an angularly stable plate and the subsequent stripping of the internal hexagon of the screw head. The self-locking of the screw-plate threaded joint was verified by calculation and the effect of the angle of the thread on the head of the locking cortical bone screw on self-locking was evaluated. The magnitude of the torque, causing the stripping of the internal hexagon (the Inbus type head) of a locking cortical bone screw with a shank diameter of 3.5 mm from Ti6Al4 V titanium alloy to ISO 5832-3, was determined experimentally. Also, it was experimentally found that the rotation of the screwdriver end with a hexagonal tip inside the locking cortical bone screw head during stripping of the internal hexagon causes strain of the screw head perimeter and thereby an increase of thread friction. The effect of tightening torque on the possibility of loosening of the locking cortical bone screw from the locking hole of an angularly stable plate was assessed experimentally. From the evaluation of five alternative shapes of locking cortical bone screw heads in terms of the acting stress and generated strains, it follows that the best screw is the screw with the Torx type head, which demonstrates the lowest values of reduced stress and equivalent plastic strain. Based on experiments and simulations the authors recommend that all global producers of locking cortical bone screws for locking holes of angularly stable plates use the Torx type heads, and not heads of the Inbus type or the Square, PH, PZ types.

## Introduction

Traumas of the skeleton are treated using trauma implants which are applied into the body permanently or until the fracture heals or requires re-operation [1–4]. Sometimes the implant is extracted, either at the patient’s request or if an undesirable reaction with the patient’s body occurs. Presently, angularly stable plates are commonly used in medical practice. These plates are most frequently used for more complicated fractures, particularly in the area of the epiphysis [3, 4].

**Fig. 1:**
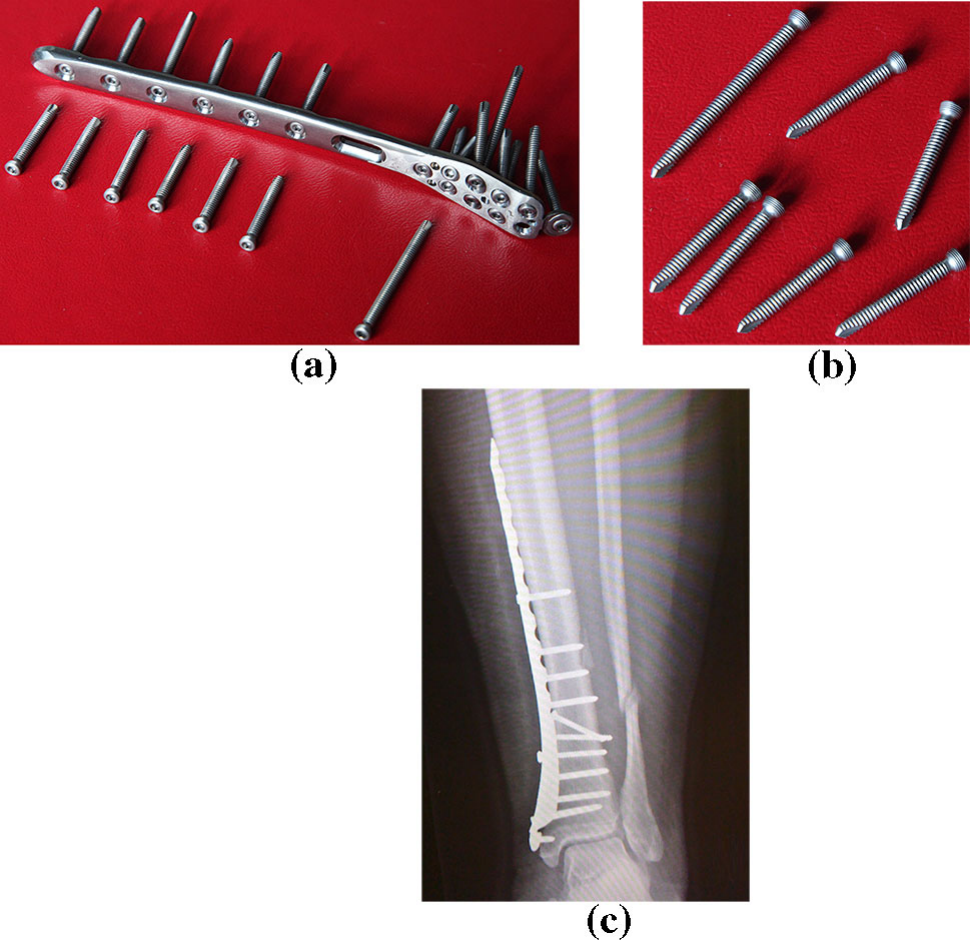
**a** Angularly stable plate with locking bone screws, **b** locking bone screws, **c** their application (photos a, b Čada)

The principle of angularly stable plates is based on the solid fixation of the screw conical head in the plate hole by the screw thread. The thread on the conical screw head must have the same or smaller pitch than the thread on the shank (bone thread). The thread on the screw head can be multi-thread. During screw tightening, bone fragments must not be compressed (or decompressed) against the plate (or from the plate). Angularly stable plates are produced as direct plates and also as shaped plates according to the anatomical shape of the bone surface [1, 3, 4] (Fig. 1).

The system of angularly stable plates is based on the internal fixator principle. An angularly stable connection of the plate system with the bone is achieved using a fixed screw-plate connection. Thereby, part of the load of the plate system is transferred from the screw-bone connection to the screw-plate connection. Therefore, the system can be used successfully even in lesser quality and osteoporotic bones.

If an angularly stable plate is used with locking screws, then the plate and screws together form a sufficiently strong and rigid system. The plate does not need to be pressed against the bone; therefore, the bone´s blood supply is not restricted. Locking screws can be applied unicortically, without compromising the strength of the system or fracture fixation, because the screws are fixed in the plate.

According to the operating procedure, the locking cortical bone screws in an angularly stable plate must be tightened manually through a torque limiter [5] with the prescribed tightening torque. For locking bone screws with a shank diameter of 3.5 mm the prescribed tightening torque is 1.5 Nm [6].

**Fig. 2:**
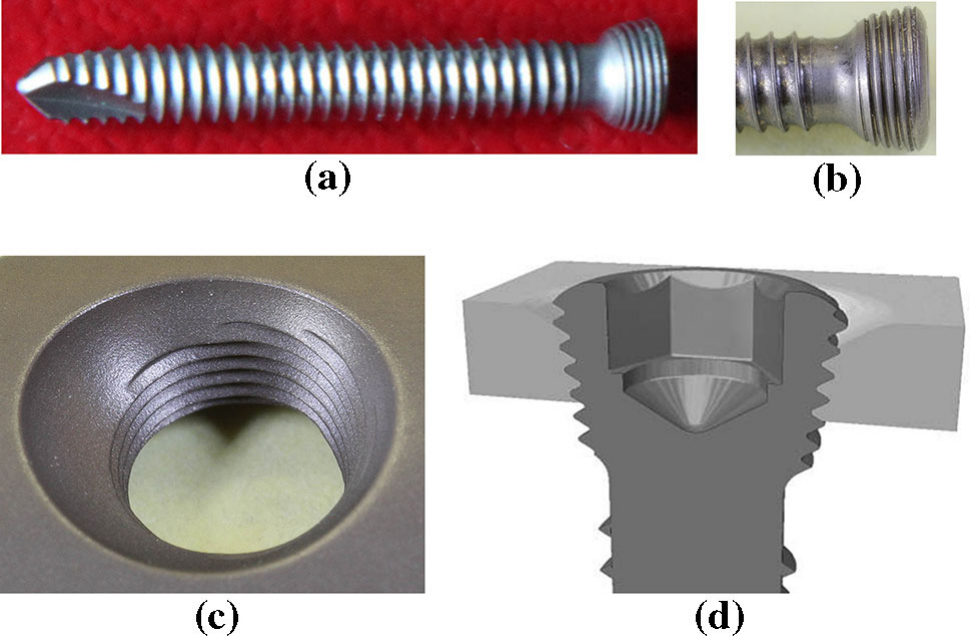
**a** Locking self-tapping bone screw, **b** detail of screw head, **c** locking hole in an angularly stable plate, **d** thread joint (photos a-c Čada)

**Fig. 3:**
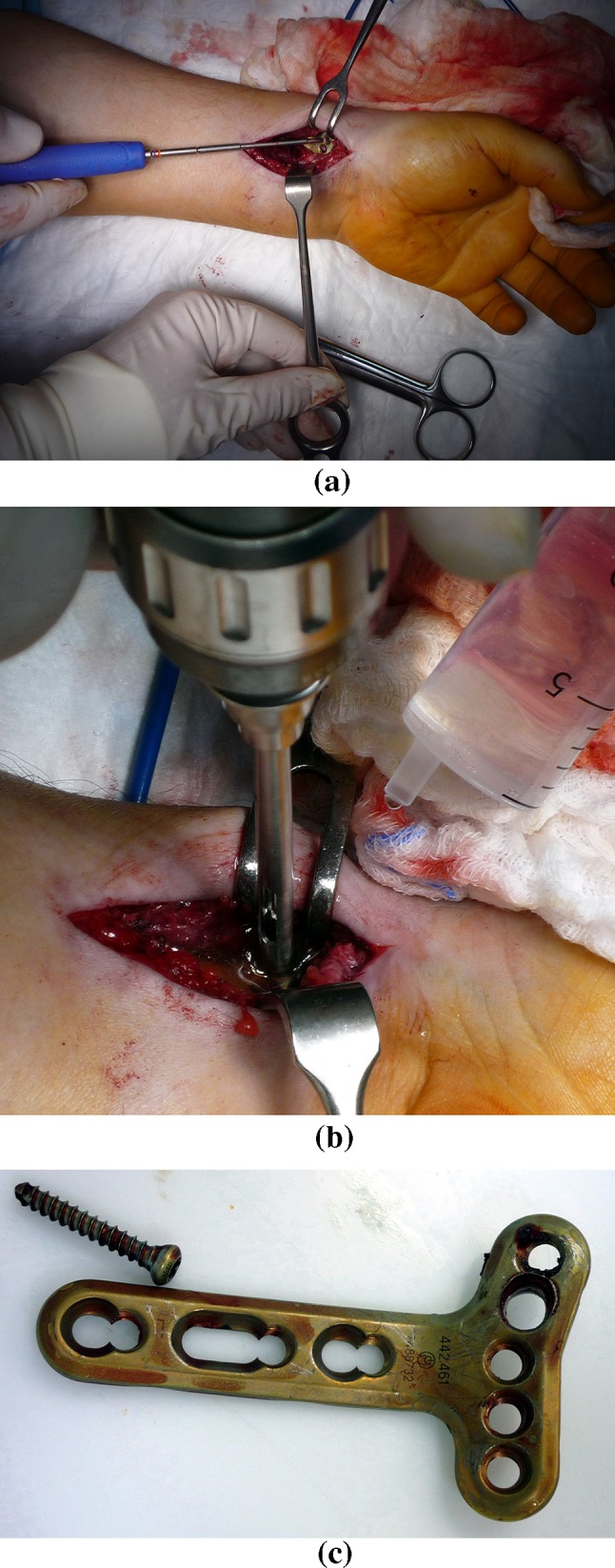
**a** Angularly stable plate fixed by locking bone screws to hand bones and the screwdriver used for their extraction, **b** drilling of the locking bone screw head on an angularly stable plate inside the hand, **c** angularly stable plate removed from the hand after the drilling of the locking bone screw head (photos Demel)

Nevertheless, even when keeping to this procedure, during the extraction of titanium-alloy implants the problems with loosening of some locking bone screws (Fig. 2) from the locking holes of an angularly stable plate often arise [7–9]. Sometimes the internal hexagon (Inbus) of the screw heads is stripped. This then requires complicated drilling of such screw heads (Fig. 3) and the subsequent removal of the screw shanks from the bone using an extraction set, which requires undesirable extension of the operating times.

### Trauma Implants Materials

Trauma implants are produced using austenitic steel 1.4441 according to ISO 5832-1 (formerly to ČSN,17,350 in the Czech Republic) and titanium alloy Ti6Al4 V according to ISO 5832-3. For both these materials the tensile strength is the same: *R*_m_ = (860 ÷ 1050) MPa. The modulus of elasticity of titanium alloy *E* = 113.5 GPa, the modulus of elasticity of stainless steel *E* = 210 GPa, so the titanium alloy is 145% more elastic. A comparison of properties of different materials can be made by the method specified in [10]. Titanium alloy is more suitable for trauma implants production than austenitic steel because in terms of biocompatibility it is more inert. Titanium alloy can be surface anodised, which is a patented procedure during which the titanium oxide is produced on the surface of the implant. The anodised layer is produced by electrolysis in an alkali or acid solution [11].

## Methods

For the analysis of locking self-taping bone screws for angularly stable plates the laboratory experiments and the numerical simulations were carried out.

The experiments (see Sect. 2.1) concern the verification of thread joint self-locking, the determination of the head perimeter before and after the stripping of the internal hexagon in the bone screw head (Inbus), the measurement of hexagon bit dimensions, the determination of torque causing the stripping of the internal hexagon in the bone screw head and the determining the effect of the tightening torque on the unscrewing of the locking cortical bone screw from the locking hole of the angularly stable plate. The experiments focus only on Inbus type screw heads which generate the most problems in medical practice (stripping).

The numerical simulations (see Sect. 2.2) were carried out by MSC.Marc/Mentat software which uses the Finite Elements Method. The influence of torque on the most commonly used screw head types—Inbus, Square, PH, PZ and Torx was simulated.

### Experiments

#### Verification of Thread Joint Self-Locking

Verification of the self-locking of the threaded joint by the locking bone screws—locking holes in angularly stable plate (Fig. 3) was done by calculation. The effect of the angle of the thread on the screw head on the self-locking was evaluated.

Self-locking is achieved according to the ratio of the thread angle *α* and friction angle *φ*. There are three possibilities:

- *α* > *φ* … non-self-locking threaded joints (the nut starts moving itself by the axial force, it is valid for multi-thread moving screw joints),
- *α* = *φ* … self-locking limit of threaded joint (equilibrium is achieved only through the effect of sliding friction in threads),
- *α* < *φ* … self-locking threaded joints (it is valid for all joining screws and single-thread moving screws, the nut cannot loosen itself spontaneously—it will not loosen),

In the case of the locking cortical bone screw in question (Fig. 2), production and subsequent measurement show the following parameters:

- thread peak angle on screw head: *β* = 60º (metric thread),
- thread span (distance between adjacent threads): *p* = 0,4 mm,
- thread pitch (distance for one rotation): *s* = 2*p* = 0,8 mm (dual-thread),
- friction angle in threads:
  $$\phi = {\text{arctg}}\;\left[ {\;\frac{f}{{\cos \,\left( {\frac{\beta }{2}} \right)}}} \right]\; = {\text{arctg}}\left[ {\frac{0,15}{{\cos \,\left( {\frac{60}{2}} \right)}}} \right]\;\;\; = { 9}. 8 2 6 4 2 9 8 1 5 { } = { 9}. 8^\circ ,$$
- sliding coefficient: *f* = 0.11–0.17,
- thread pitch angle: *α* ≤ 3.3º.

The result is shown in Sect. 3.

#### Measurement of Diameter of Heads of Tested Screws with the Internal Hexagon (Inbus)

**Fig. 4:**
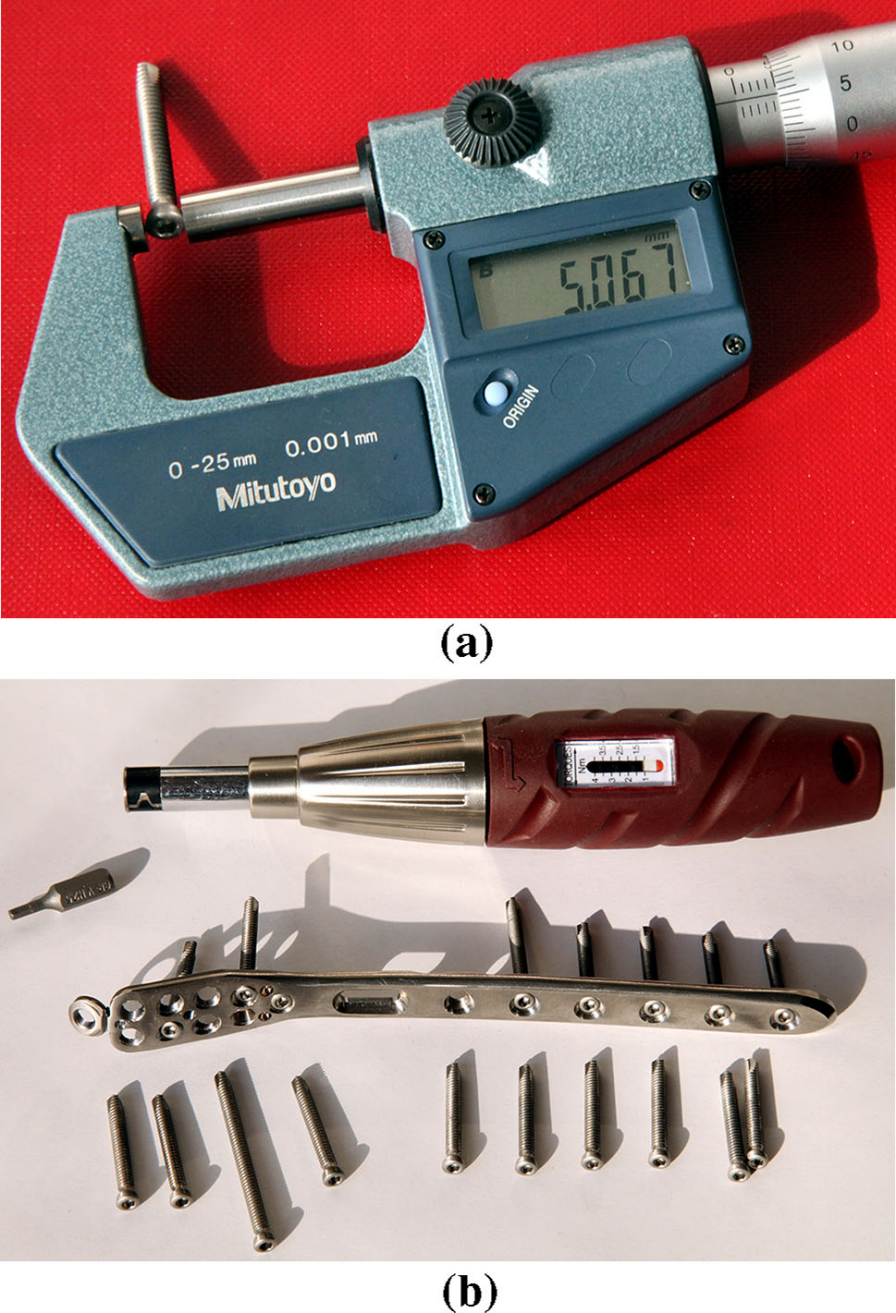
**a** Measurement of bone screw head diameter using a Mitutoyo digital micrometer with a range of (0–25) mm, **b** torque screwdriver with a 2.5 mm bit and angularly stable plate with locking bone screws (photos Čada)

The diameter of the head of each tested locking bone screw with a shank diameter of 3.5 mm from titanium alloy Ti6Al4 V according to ISO 5832-3 was measured 10 times at various points while rotating the screw using a Mitutoyo digital micrometer with the range of (0–25) mm, accuracy 0.001 mm, made in Japan, see Fig. 4a and subsequently the mean values and the absolute measurements errors were calculated (see Table 1).

**Table 1:** Measured values of diameter of the Inbus type head of locking bone screw with a shank diameter of 3.5 mm from Ti6Al4 V titanium alloy according to ISO 5832-3 before and after the stripping of the internal hexagon in the screw head and the calculated mean value and absolute error of measurement – for screws 1 to 5

Measurement no.	Screw no. with the Inbus type head									
	1	1 after the stripping of hexagon by torque of 2.5 Nm	2	2 after the stripping of hexagon by torque of 3.1 Nm	3	3 after the stripping of hexagon by torque of 3.1 Nm	4	4 after the stripping of hexagon by torque of 3.2 Nm	5	5 after the stripping of hexagon by torque of 3.2 Nm
1	5.010	5.036	5.013	5.089	5.003	5.086	5.030	5.101	5.006	5.103
2	5.007	5.059	5.044	5.059	5.016	5.054	5.044	5.098	5.014	5.098
3	5.044	5.062	5.047	5.058	5.039	5.094	5.007	5.053	5.039	5.055
4	5.051	5.024	5.003	5.097	4.999	5.054	5.041	5.093	5.007	5.061
5	5.005	5.025	5.010	5.068	5.001	5.052	5.045	5.078	5.049	5.069
6	5.014	5.057	5.043	5.060	5.006	5.091	5.004	5.067	5.011	5.089
7	5.035	5.074	5.019	5.101	5.035	5.074	5.005	5.080	5.049	5.060
8	5.006	5.036	5.024	5.112	5.002	5.059	5.036	5.060	5.055	5.099
9	5.000	5.038	5.040	5.077	5.014	5.047	5.016	5.096	5.004	5.094
10	5.033	5.076	5.002	5.098	5.000	5.064	5.005	5.068	5.041	5.064
Mean value (mm)	5.021	5.049	5.025	5.082	5.012	5.068	5.023	5.079	5.028	5.079
Absolute error (mm)	0.016	0.017	0.015	0.018	0.012	0.015	0.016	0.014	0.019	0.017

#### Measurement of Hexagon Bit Dimensions

**Fig. 5:**
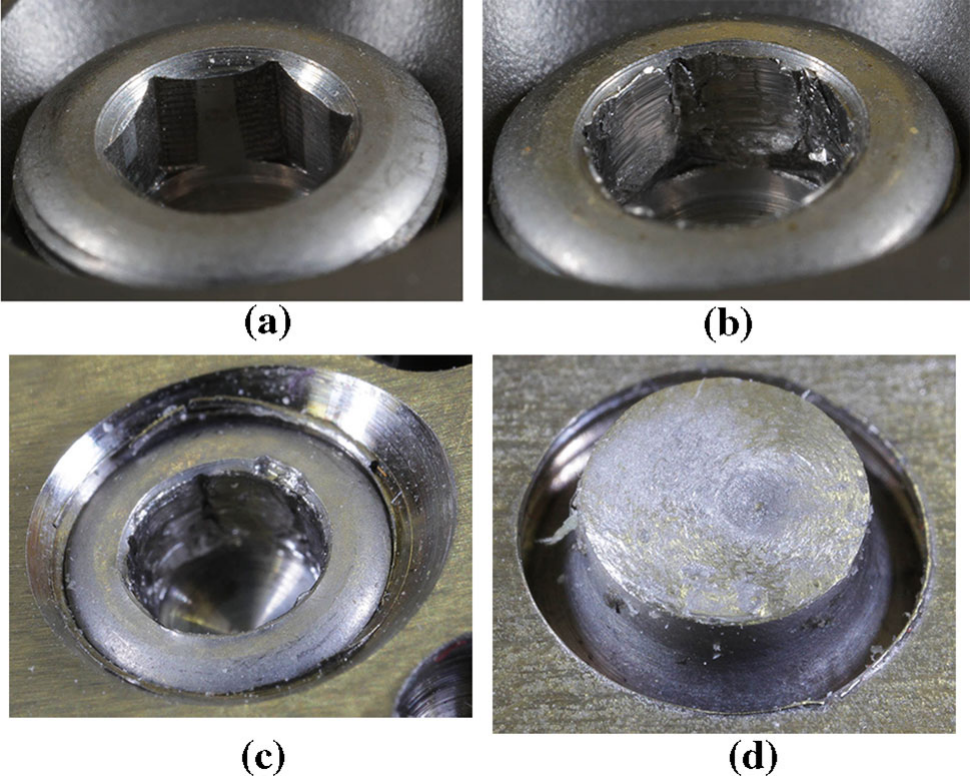
Internal hexagon in the head of a locking self-tapping bone screw **a** before, **b** after using a screwdriver, **c** stripped internal hexagon in the screw head after using a screwdriver, **d** fracture surface after the fracture of the screw shank (photos Čada)

A KRAFTWERK torque screwdriver—model 2039, range (1–4) Nm ± 6.0%, according to ISO 6789 (Fig. 5), which was supplied with a calibration certificate, was used for the experiments. The distance of the opposite walls of the 2.5 mm hexagonal bit, which was used with the torque screwdriver for experiments, was measured 10 times (rotating the bit) using a Mitutoyo digital micrometer with a range of (0–25) mm, accuracy 0.001 mm, made in Japan (Fig. 8). The mean value 2.489 mm and the absolute measurement error 0.001 mm were calculated from the obtained results.

#### Determination of Torque Causing Stripping of Internal Hexagon in the Bone Screw Head (Inbus)

The magnitude of the torque causing stripping of the internal hexagon in the locking cortical bone screw head was determined experimentally in the laboratory. The locking bone screw was fixed with a strong clamping force by its shank in a bench vice to prevent its rotation during the application of the torque screwdriver. A torque screwdriver with a 2.5 mm hexagonal bit was used for the experiment (see Sect. 2.1.3).

Using its dial setting, the torque screwdriver was gradually set to tightening torque from 1 Nm (for screw 1), or 2 Nm (for screws 2 and 3) respectively, upwards in 0.1 Nm increments. Then the screwdriver was rotated manually with a 2.5 mm bit in the locking bone screw head. With the low torques set, the internal limiter of the screwdriver always engaged which indicated that at that particular torque setting the internal hexagon in the bone screw head was not stripped, see Fig. 5a. After the stripping of the internal hexagon by the bit of the torque screwdriver, the dial of the torque screwdriver showed torque values—for screw No. 1 it was 2.5 Nm, for screw No. 2 it was 3.1 Nm and for screw No. 3 it was also 3.1 Nm (see Table 1).

A comparison of the measured values of mean diameter of the Inbus type head of locking bone screw with a shank diameter of 3.5 mm from Ti6Al4 V titanium alloy according to ISO 5832-3 before and after the stripping of the internal hexagon in the screw head and the calculated mean value and the absolute error of measurement—for screws 1–5 is seen in Table 2.

**Table 2:** Comparison of measured values of mean diameter of the Inbus type head of locking bone screw with a shank diameter of 3.5 mm from Ti6Al4 V titanium alloy according to ISO 5832-3 before and after the stripping of the internal hexagon in the screw head and the calculated mean value and absolute error of measurement—for screws 1–5

Locking bone screw with a shank diameter of 3.5 mm	Screw No. with the Inbus type head				
	1	2	3	4	5
Mean value of screw head diameter and its absolute error (mm)	5.021 ± 0.016	5.025 ± 0.015	5.012 ± 0.012	5.023 ± 0.016	5.028 ± 0.019
Torque at which the internal hexagon on the screw head was stripped (Nm)	2.5	3.1	3.1	3.2	3.2
Mean value of screw head diameter and its absolute error after the stripping of the internal hexagon in the screw head (mm)	5.049 ± 0.017	5.082 ± 0.018	5.068 ± 0.015	5.079 ± 0.014	5.079 ± 0.017
Difference in mean values of screw head diameter before and after the stripping of the internal hexagon (mm)	0.028	0.057	0.056	0.056	0.051

The results are commented on in Sect. 3.

#### Determination of the Head Perimeter Strain after the Stripping of the Internal Hexagon in the Bone Screw Head (Inbus)

It is also important to determine whether the rotation of the end of the screwdriver with a 2.5 mm hexagonal bit inside the locking cortical bone screw head, during the stripping of the internal hexagon, causes greater strain of the screw head perimeter. Greater strain of the screw head leads to an undesirable increase of friction of the thread in the locking hole of the angularly stable plate. Therefore, the diameter of each locking bone screw head (see Table 2) was measured 10 times at various points while rotating the screw, using a Mitutoyo digital micrometer with a range of (0–25) mm, accuracy 0.001 mm, made in Japan. Subsequently the mean value and the absolute measurement error were calculated.

A comparison of the measured values of the mean diameter of the locking bone screw with a shank diameter of 3.5 mm from titanium alloy Ti6Al4 V according to ISO 5832-3 before and after the stripping of the internal hexagon in the screw head, see Fig. 5c and a comparison of the absolute measurement errors were made.

#### Determining the Effect of the Tightening Torque on the Unscrewing of the Locking Cortical Bone Screw from the Locking Hole of the Angularly Stable Plate (Inbus)

To determine the effect of tightening torque on the unscrewing of a locking cortical bone screw from the locking hole of the angularly stable plate, the laboratory experiments were performed using the KRAFTWERK torque screwdriver—model 2039/3778, range (1–4) Nm (±6,0%) according to ISO 6789. On the screwdriver dial, torque values were set from 0.1 Nm upwardly in 0.1 Nm increments. A 2.5 mm hexagonal bit was used for experiments. The plate in question was gripped in a workbench vice.

First, the locking cortical bone screws with a shank diameter of 3.5 mm and with the Inbus type heads were screwed into the locking holes of an angularly stable plate by the torque screwdriver at the set torque. Subsequently the screws were unscrewed using the torque screwdriver. During loosening of the bone screws from the holes of the angularly stable plate the dial of the torque screwdriver was initially set to lower torque values than the torque necessary to screw in the given screw, and subsequently the torque was increased in 0.1 Nm increments until the screw was released and unscrewed.

The measured torque values for respective screws with the Inbus type heads in specific locking holes of an angularly stable plate are shown in Tables 3 and 4.

**Table 3a:** Measured values of torques at which the locking bone screw with a shank diameter of 3.5 mm and with the Inbus type head from Ti6Al4 V titanium alloy according to ISO 5832-3 was loosened from the angularly stable plate in relation to the previously used tightening torque—for screw No. 6

Locking bone screw with a shank diameter of 3.5 mm	Screw No. 6											
Measurement no.	1	2	3	4	5	6	7	8	9	10	11	12
Marking of used hole in plate	11	11	11	11	11	11	11	11	11	11	11	11
Used tightening torque (Nm)	0.1	0.2	0.3	0.4	0.5	0.6	0.7	0.8	0.9	1.0	1.1	1.2
Time delay before loosening of the screw (min.)	1	1	1	1	1	1	1	1	1	1	1	1
Torque at which the screw was loosened from the hole in the plate (Nm)	0.1	0.3	0.4	0.4	0.5	0.7	0.9	0.9	1.0	1.1	1.2	1.3
Note	–	–	–	–	–	–	–	–	–	–	–	–

**Table 3b:** Measured values of torques at which the locking bone screw with a shank diameter of 3.5 mm and with the Inbus type head from Ti6Al4 V titanium alloy according to ISO 5832-3 was loosened from the angularly stable plate in relation to the previously used tightening torque—for screw No. 6

Locking bone screw with a shank diameter of 3.5 mm	Screw No. 6										
Measurement no.	13	14	15	16	17	18	19	20	21	22	23
Marking of used hole in the plate	11	11	11	11	11	11	11	11	11	11	11
Used tightening torque (Nm)	1.3	1.4	1.5	1.6	1.7	1.8	1.9	2.0	2.1	2.2	2.3
Time delay before loosening of the screw (min)	1	1	1	1	1	1	1	1	1	1	1
Torque at which the screw was loosened from the hole in the plate (Nm)	1.4	1.4	1.5	1.6	1.7	1.8	1.9	2.0	2.2	2.2	2.3
Note	–	–	–	–	–	–	–	–	–	–	During loosening, the internal hexagon was deformed and the bit remained stuck; not stripped

**Table 4:** Measured values of torques at which the locking bone screw with a shank diameter of 3.5 mm and with the Inbus type head from Ti6Al4 V titanium alloy according to ISO 5832-3 was loosened from the angularly stable plate in relation to the previously used tightening torque—for screw No. 7

3.5 mm locking bone screw	Screw No. 7						
Measurement no.	1	2	3	4	5	6	7
Marking of used hole in the plate	10	10	10	10	10	10	10
Used tightening torque (Nm)	1.0	1.1	1.2	1.3	1.4	1.5	1.6
Time delay before the loosening of the screw (min)	1	1	1	1	1	1	1
Torque at which the screw was loosened from the hole in the plate (Nm)	1.0	1.2	1.3	1.3	1.5	1.7	
Note	Minor strain of the internal hexagon	–	–	–	Major strain of the internal hexagon	–	–

The results are commented on in Sect. 3.

### Numerical Simulations

The evaluation of various head shapes of locking cortical bone screws with respect to the acting stress and produced strains was carried out by the numerical simulations.

**Fig. 6:**
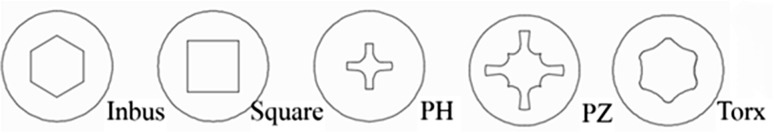
Geometry of most commonly used screw heads

A head geometry was found for the most commonly used screw head types – Inbus, Square, PH, PZ and Torx (Fig. 6), and this geometry was subsequently designed in Autodesk AutoCAD 2016 software. The created models were then exported to MSC.Marc/Mentat software, where the screws were subject to the Finite Elements Method (FEM) analysis. A total of five calculations simulating tightening and loosening of screws (Fig. 6) were performed.

#### Setting of the FEM Analysis

During screwing, the rotating movement of the screwdriver (rotating around its longitudinal axis) with the screw head exists. In the FEM analysis the screwdriver was defined as an absolutely rigid (non-deformable) body which is an acceptable simplification of the real state. A rotational velocity of 0.0873 rad s^−1^ (corresponds to 5 s^−1^) was prescribed to the screwdriver. The screw head was defined as a real (deformable) body. Friction between both bodies was set by the Coulomb friction coefficient *f* = 0.05.

The boundary condition of the absolutely rigid rotating body will, to a certain extent, distort the real values of reduced stress, nevertheless, for comparison of the various screw head shapes (monitoring the trend of the comparison of stress and plastic strain values) the utilization of this calculation model is suitable.

The screw head was fixed at its external edge, i.e. outer radius, in all directions, which corresponds to rigid fixation (screw head is fixed in plate). In all models the finite elements grid was made finer in areas of expected high values of stress and plastic strain. The finite elements grid was formed by type 11 elements (four-node element in MSC.Marc/Mentat software). All analyses (5 calculations for various screw heads) were set identically.

Reduced stress according to the HMH (von Mises) theory, equivalent plastic strain and the relation between the tightening torque and screwdriver torsion angle were analysed in the screw heads.

Analyses were calculated simply—as 2D calculations (planar strain).

The screw head material was selected to be an alloy called “asm_Tita 79”, which is included in material database of the MSC.Marc/Mentat software. The alloy has the following mechanical properties: yield strength *R*_e_ = 450 MPa, ultimate strength *R*_m_ = 1000 MPa, modulus of elasticity *E* = 110 GPa, fatigue strength (10^7^ cycles) *σ*_f_ = 289 MPa, hardness to Brinell *HB* = 225. This material is very similar to the real material of tested screws (see Sect. 1.1). According to the fact that the aim of the simulations was the reciprocal comparison of various screw heads at similar loading, the use of this material for simulations did not affect the general results of comparison.

## Results

### Results of the Experiments

Condition c) is fulfilled (see Sect. 2.1.1); therefore, the threaded joint by the locking bone screws—locking holes in the angularly stable plate (Fig. 2) is a self-locking threaded joint. To reduce self-locking, it would be necessary to increase the thread pitch angle on the screw head.

The measured values of diameter of the Inbus type head of the locking bone screw with a shank diameter of 3.5 mm from Ti6Al4 V titanium alloy according to ISO 5832-3 (see Sect. 2.1.2) and calculated mean values and absolute errors of measurements for ten screws is seen in Table 1.

The measured values of distance of the opposite walls of the 2.5 mm hexagonal bit which was used with the torque screwdriver during experiments and the calculated mean value and absolute error of ten measurements is seen in Sect. 2.1.3.

The measured values of diameter of the Inbus type head of the locking bone screw with a shank diameter of 3.5 mm from Ti6Al4 V titanium alloy according to ISO 5832-3 before and after the stripping of the internal hexagon in the screw head (see Sect. 2.1.4) and the calculated mean value and the absolute error of measurement—for screws 1 to 5 is seen in Table 1.

The experiments (see Sect. 2.1.4) showed that during the increase of the torque up to the strip limit of the internal hexagon the locking bone screw head is subject to the plastic strain of the internal hexagon—undesirable strain, see Fig. 5b. The existence of plastic strains is evident after release, when the hexagonal bit is pressed into the head so much that it is difficult to remove it. The internal hexagon in the locking bone screw head is stripped either by the repeated action of lower torque and gradual accumulation of plastic strains, or by the increased torque set on the torque screwdriver.

Table 2 shows that for screw 1, the internal hexagon in the locking bone screw head (see Sect. 2.1.4) was stripped already at a torque of 2.5 Nm, whereas for screws 2 and 3 the torque was 3.1 Nm. In the case of screw 1, the effect of the wear of the internal hexagon by small plastic strains was evident because during this experiment the torque on the screwdriver´s dial was set already from 1 Nm upwardly in 0.1 Nm increments, whereas for screws 2 and 3 it was set in 0.1 Nm increments from 2 Nm. After setting the torque on the torque screwdriver dial the screwdriver handle was rotated manually with a 2.5 mm bit inserted in the locking bone screw head. With the low torques set, the internal limiter of the screwdriver always engaged which indicated that at that particular torque setting the internal hexagon in the bone screw head was not stripped. At higher torque values the internal hexagon in the bone screw head was worn by plastic strains caused by the edges of the hexagonal bit.

From Table 2 it is evident that the rotation of the screwdriver end with a hexagonal bit in the locking cortical bone screw head during stripping of the internal hexagon (see Sect. 2.1.5) causes a slight increase of the bone screw head diameter. During use of the locking cortical bone screw in the locking hole of the angularly stable plate, the internal hexagon in the screw head is stripped when the torque is exceeded. This directly relates to the increase in the screw head diameter by plastic strain with a subsequent increase of the friction forces acting in the thread of the locking hole of the angularly stable plate.

By determining the effect of tightening torque on the unscrewing of the locking cortical bone screw from the locking hole of the angularly stable plate (see Sect. 2.1.6), it was found that the tightening of the locking bone screw with a shank diameter of 3.5 mm in the locking hole of the angularly stable plate with a tightening torque of 0.1 Nm already provides for a very strong threaded joint.

The measured values of torques at which the locking bone screw with a shank diameter of 3.5 mm and with the Inbus type head from Ti6Al4 V titanium alloy according to ISO 5832-3 was loosened from the angularly stable plate in relation to the previously used tightening torque—for screw No. 6 are shown in Table 3, for screw No. 7 they are shown in Table 4.

Using a 10× magnifying glass, it was observed that during repeated strain of the internal hexagon by screwing and unscrewing of the same locking bone screw into the locking hole of an angularly stable plate, flakes of metal are produced on the walls of the internal hexagon in places where the hexagonal bit acts. These flakes gradually peel off the walls of the internal hexagon and after removal of the bit they fall to the bottom of the internal hexagon by gravity. This means, that during operation these metal flakes can contaminate the human body.

### Results of the Simulations

The results of the simulations are shown in the following five Sects. 3.2.1–3.2.5. Every section shows the results for one of five most commonly used screw head types—Inbus, Square, PH, PZ and Torx.

#### Results of the Analysis of Screw with the Inbus Type Head

The maximum reduced stress values were found in the points of the expected maximum, i.e. in areas of contact (mechanical contact) between the rigid body (screwdriver) and deformed body (screw head).

**Fig. 7:**
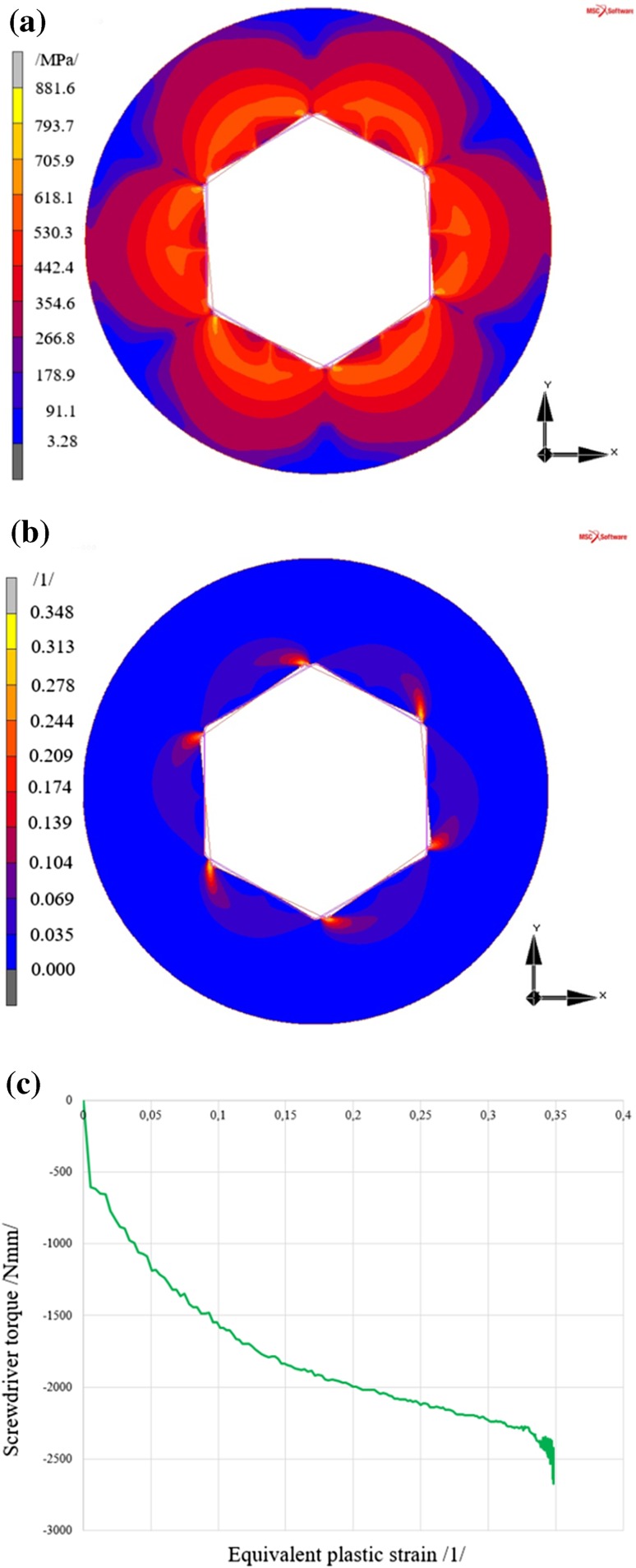
Results of the analysis of the screw with the Inbus type head **a** reduced stress according to HMH (von Mises) theory, **b** equivalent plastic strain in the screw head, **c** development of equivalent plastic strain in the most stressed area of the screw

Rotation of the screwdriver by 5° generates a reduced stress in the screw head of 881.6 MPa, see Fig. 7a, according to the HMH (von Mises) theory, where the resultant equivalent plastic strain, see Fig. 7b, reaches 0.348.

Figure 7c shows the relation between the equivalent plastic strain in the area of maximum plastic strain and the screwdriver torque. It is evident that plastic deformation occurs almost immediately after the action of the rigid body (screwdriver).

A discussion of the results is shown in Sect. 4.

#### Results of the Analysis of Screw with the Square Type Head

The maximum reduced stress value again occurs in the area of the expected maximum, i.e. in the area of contact of the rigid body (screwdriver) with the deformable body (screw head).

**Fig. 8:**
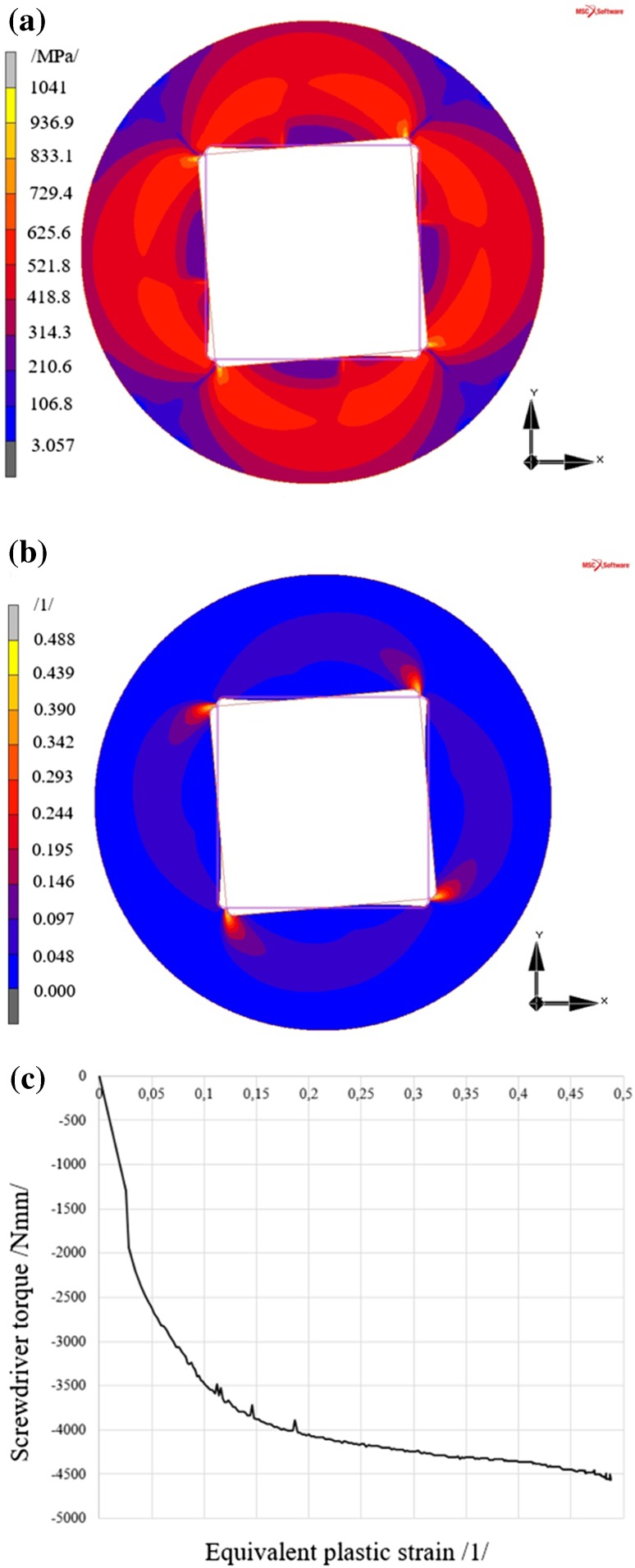
Results of the analysis of the screw with the Square type head **a** reduced stress according to HMH (von Mises) theory, **b** equivalent plastic strain in the screw head, **c** development of equivalent plastic strain in the most stressed area of the screw

Rotation of the screwdriver by 5° generates a reduced stress in the screw head of 1041 MPa, see Fig. 8a, according to the HMH (von Mises) theory, where the resultant equivalent plastic strain, see Fig. 8b, reaches 0.485.

Figure 8c shows the relation between the screwdriver torque and equivalent plastic strain in the area of maximum plastic strain and the screwdriver torque. In the screw with the Square type head the plastic strain occurs almost immediately after action of the screwdriver in the screw head.

A discussion of the results is shown in Sect. 4.

#### Results of the Analysis of Screw with the PH Type Head

The maximum reduced stress value again occurs in the area of the expected maximum, i.e. in the area of contact of the rigid body (screwdriver) with a deformable body (screw head).

**Fig. 9:**
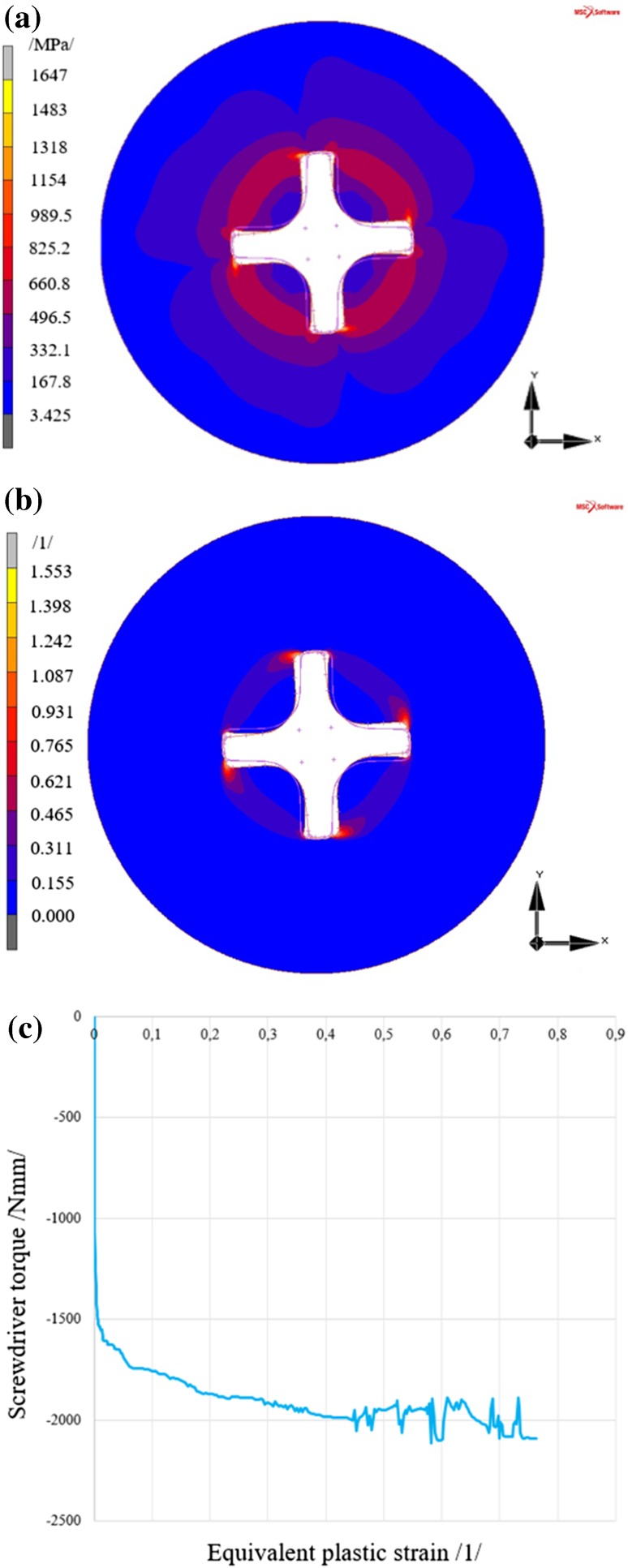
Results of the analysis of the screw with the PH type head **a** reduced stress according to HMH (von Mises) theory, **b** equivalent plastic strain in the screw head, **c** development of equivalent plastic strain in the most stressed area of the screw

Rotation of the screwdriver by 5° generates a reduced stress in the screw head of 1647 MPa, see Fig. 9a, according to the HMH (von Mises) theory, where the resultant equivalent plastic strain, see Fig. 9b, reaches 0.762.

Figure 9c shows the relation between the equivalent plastic strain in the area of maximum plastic strain and the screwdriver torque. Plastic strain in the screw with the PH type head occurs at about 1400 Nmm.

A discussion of the results is shown in Sect. 4.

#### Results of the Analysis of Screw with the PZ Type Head

**Fig. 10:**
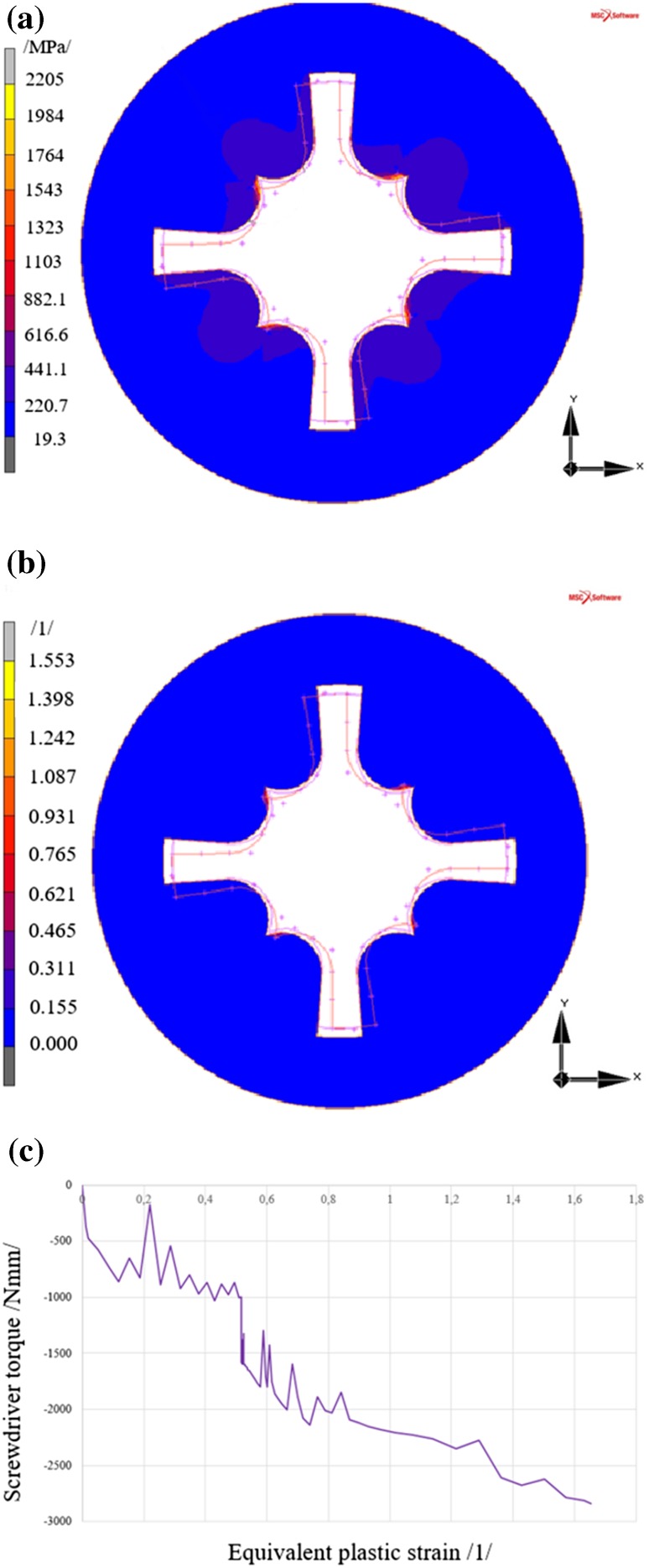
Results of the analysis of the screw with the PZ type head **a** reduced stress according to HMH (von Mises) theory, **b** equivalent plastic strain in the screw head, **c** development of equivalent plastic strain in the most stressed area of the screw

The maximum reduced stress value again occurs in the area of presence of short arms of the screw, see Fig. 10a, in areas of contact of the rigid body (screwdriver) with the deformable body (screw head).

Rotation of the screwdriver by 5° generates a reduced stress in the screw head of 2205 MPa, see Fig. 10a according to the HMH (von Mises) theory, where the resultant equivalent plastic strain, see Fig. 10b, reaches 1.242.

Figure 10c shows the relation between the equivalent plastic strain in the area of maximum plastic strain and the screwdriver torque.

A discussion of the results is shown in Sect. 4.

#### Results of the Analysis of Screw with the Torx Type Head

The maximum reduced stress value again occurs in the area of the expected maximum, i.e. the area of contact of the rigid body (screwdriver) with a deformable body (screw head).

**Fig. 11:**
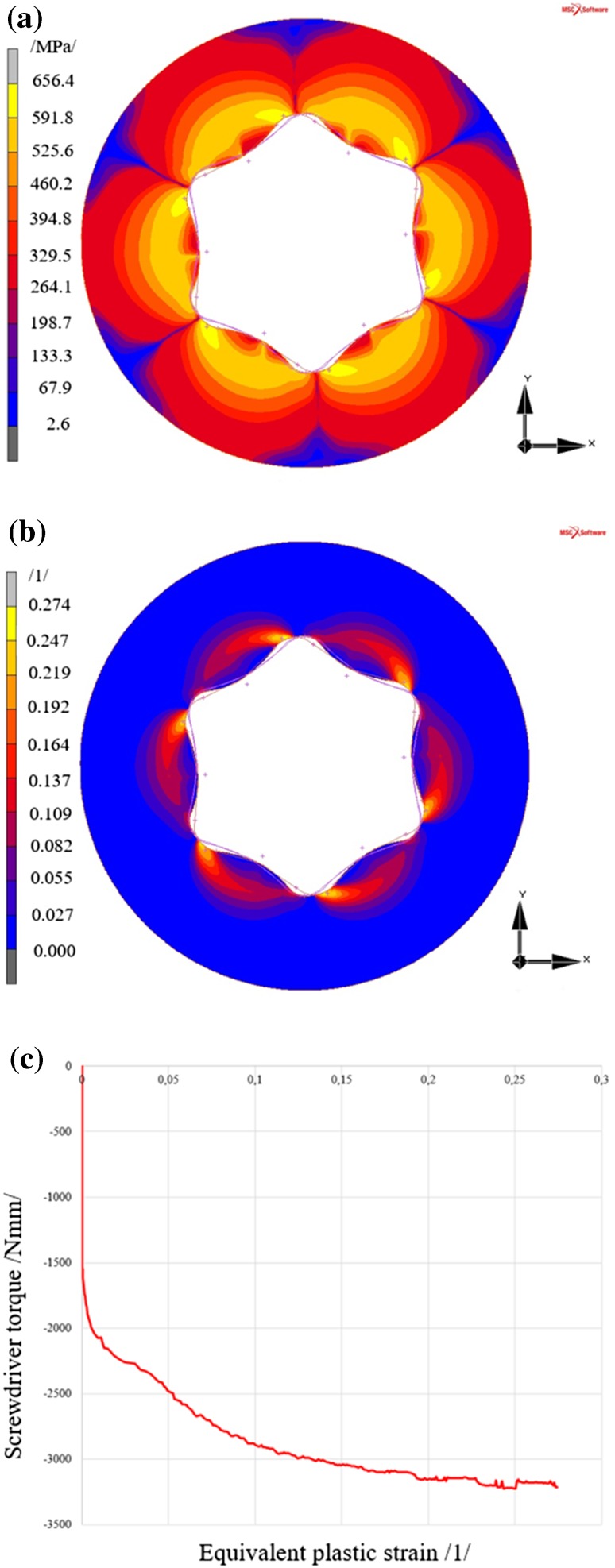
Results of the analysis of the screw with the Torx type head **a** reduced stress according to HMH (von Mises) theory, **b** equivalent plastic strain in the screw head, **c** development of equivalent plastic strain in the most stressed area of the screw

Rotation of the screwdriver by 5° generates a reduced stress in the screw head of 656.4 MPa, see Fig. 11a, according to the HMH (von Mises) theory, where the resultant equivalent plastic strain, see Fig. 11b, reaches 0.274.

Figure 11c shows the relation between the equivalent plastic strain in the area of maximum plastic strain and screwdriver torque. Compared to the Inbus type head screw (Fig. 7c), plastic strain in the screw with the Torx type head occurs at about 1600 Nmm.

With respect to lower values of stress and strain the Torx type head is better than the Inbus type head.

A discussion of the results is shown in Sect. 4.

## Discussion

Verifying of the self-locking of the threaded joint by calculation and evaluation of the effect of thread pitch on the locking cortical bone screw head on self-locking was carried out (see Sect. 2.1.1).

The magnitude of torque causing the stripping of the internal hexagon (Inbus type head) of the locking cortical bone screw with a shank diameter of 3.5 mm from titanium alloy Ti6Al4 V to ISO 5832-3 was experimentally determined (see Sect. 2.1.4).

It was experimentally determined that the rotation of the screwdriver end with a hexagonal bit inside the head of a locking cortical bone screw during stripping of the internal hexagon (Inbus type head) causes strain of the screw head perimeter and thereby increased thread friction (see Sect. 2.1.5).

In practice, doctors often use a torque limiter to tighten locking cortical bone screws in the locking holes of angularly stable plates and only estimate the tightening torque. If they tighten the locking bone screws with higher tightening torque, the screws cannot be loosened during later extraction because the higher torque needed to loosen them causes the stripping of the internal hexagon in the screw head (see Sect. 2.1.6). Then the doctor has to perform complicated drilling of the striped screw heads and subsequently remove the screw shanks from the bones using an extraction set, which causes an undesirable extension of the operating time and also additional damage of the patient´s bones (potential infection and treatment complications).

The results of the numerical analyses of alternative screw head shapes (see Sect. 3.2) were compared.

The rapid changes in relations, see mainly Figs. 9c and 10c, are related to the variable mechanical contact between the screwdriver and screw head and also to the development of plastic strain. All this affects the friction force magnitude during calculation.

From the FEM analysis results it is evident that during screwing using a screwdriver the lowest stress is generated in the screw with the Torx type head (stress is approximately 25.5% lower than in screws with the Inbus type head); also the equivalent plastic strain is lowest from the analysed heads (plastic strain is approximately 21.3% lower than in screws with the Inbus type head).

The Inbus type head of the locking cortical bone screw was analysed experimentally and also numerically (by simulations using the Finite Elements Method). Very similar results were achieved by both approaches (arising of plastic strain in the most stressed area of the screw head when loading by torque) so it is possible to state that the results are reliable for the Inbus type head.

The Inbus and the Torx type heads of the locking cortical bone screws are usually used in medical practice (traumatology, ortopaedics) and so it is not necessary to test their utilization clinically.

The remaining head types (Square, PH and PZ) of the locking cortical bone screws are used in technical practice (e.g. in mechanical engineering) but not in medicine. However the Square, PH and PZ type heads of the locking cortical bone screws are used in this paper only as alternatives for the comparison. The numerical simulations of the Square, PH and PZ type heads of the locking cortical bone screws which show worse results than the Inbus and the Torx type heads of the locking cortical bone screws support the right idea of not using them in medical practice.

Because a sufficiently good sameness of the experimental and the numerical results for the Inbus type head of the locking cortical bone screw was achieved it is possible to state that the numerical simulation of other head types (Square, PH, PZ and Torx) are also sufficiently exact even without experiments.

The highest stress and plastic strain is generated in the screw with the PZ type head.

Therefore, the best choice is to use screws with the Torx type head, which reaches the lowest values of reduced stress and also the smallest equivalent plastic strain (see Table 5).

**Table 5:** Calculated values of reduced stress according to HMH (von Mises) and equivalent plastic strain for various head types of screws from material “asm_Tita 79” in MSC.Marc/Mentat software

Screw head type	Inbus	Torx	Square	PH	PZ
Reduced stress to HMH (MPa)	881.6	656.4	1041	1647	2205
Equivalent plastic strain (–)	0.348	0.274	0.488	0.762	1.242
Evaluation		Best			Worst

The main aim of the work was not to calculate the exact values of the stress or plastic strain for alternative screw head shapes, but more likely to compare the resistance towards destruction of the screw head (screwdriver slip in the screw head). From the results, it decidedly follows that the Torx type head is the most suitable for medical practice, the Inbus type head is acceptable for medical practice and the Square, PH and PZ type heads are unsuitable for medicine practice.

**Fig. 12:**
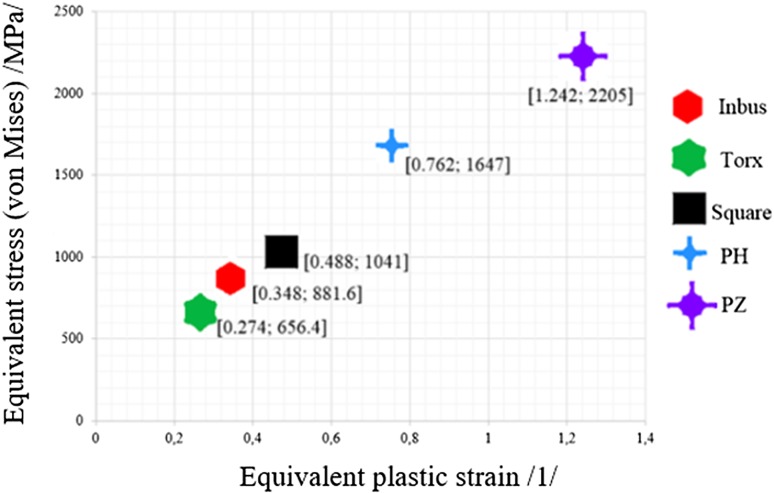
Comparison of the results of analyses of alternative screw head shapes by reduced stress according to the HMH (von Mises) theory and according to the equivalent plastic strain in the screw head

Figure 12 shows a comparison of the results of the respective analyses. The optimal screw head shape should approach the bottom left corner of the graph in Fig. 12 as closely as possible.

## Conclusion

The effect of tightening torque on the possibility of unscrewing of a locking cortical bone screw from a locking hole of an angularly stable plate was evaluated experimentally. It was found that when the locking bone screw with a shank diameter of 3.5 mm is tightened with a tightening torque of 0.1 Nm in a locking hole of an angularly stable plate, the threaded joint is very strong—without any clearance. The results of experiments (see Sect. 2.1.6) shows that for locking bone screws with a shank diameter of 3.5 mm it would be suitable to reduce the tightening torque from the current value of 1.5 Nm, prescribed in the operating procedure, to a lower value.

Based on the evaluation of five alternative head shapes of locking cortical bone screws with respect to the acting stress and generated plastic strains by simulations in MSC.Marc/Mentat software (see Sect. 3.2), the best head shape is the Torx type head. The screw with the Torx type head provides the lowest reduced stress values and also the lowest equivalent plastic strain values. Also, the screw with the Torx type head guarantees better transmission of torques even with only partial insertion of the screwdriver bit into the head compared to the Inbus type head.

Based on experiments and simulations the paper´s authors recommend that all global producers of locking cortical bone screws for locking holes in angularly stable plates use the Torx type heads and not heads of the Inbus or the Square, PH, PZ types.
